# Supplementary material for: Modeling Sequence-Space Exploration and Emergence of Epistatic Signals in Protein Evolution
Source: Mol Biol Evol. 2021 Nov 9;39(1):msab321. doi: 10.1093/molbev/msab321 (PMC8789065; doi:10.1093/molbev/msab321)
Supplement: msab321_Supplementary_Data [file msab321_supplementary_data.zip › reply_to_reviewers.pdf]

We thank both reviewers for their careful reading and positive evaluation of our manuscript. We have revised our work following the suggestions point by point, please see the detailed answers below, and the manuscript pdf with highlighted changes. We sincerely hope that both reviewers are satisfied with our revision.

The original comments by the reviewers are given in black, our replies in blue.

Reviewer: 1

This manuscript deals with the idea of developing protein sequence evolutionary models that are data-driven and consistent with experimental evolution. This model is based on a body of work that uses statistical physics and estimation concepts to model the multi-variate probability distribution of sequences in a family of proteins and whose parameters are predictive of epistatic interactions useful for contact prediction, fitness and amino acid coevolution. In particular, this article introduces a modification of the Sequence Evolution with Epistatic Contribution (SEEC) model to allow mutational events at the nucleotide scale, a control over the sequencing depth of an evolved library as well as a parameter that controls the strength of selection. This non-trivial advancement to the SEEC model is significant to quantitatively describe non-directed experimental evolution of two proteins and explain why these models had distinct efficiencies when used to generate data to perform structure prediction on these systems. The authors conclude that enough variability and a larger number of sequences is needed as well as a slightly lower selection pressure could have improved their goal of structure prediction. The authors also describe potential uses of this methodology to fine tune experiments and discuss the possibility of improving on such models to account for even more realistic experimental scenarios.

In my opinion, this article is not only exciting but has a unique fundamental contribution to the study of sequence evolution. Although the use of MSAs and Direct Coupling Analysis has already made multiple connections with experimental observables, including the group's result on generating in vivo functional sequences from such global models, it is really exciting to see a direct quantitative connection to laboratory evolution. While the SEEC model connected with statistical properties of many models of evolution and highlighted the importance of epistatic contributions, the work by Bisardi et al. makes a strong and concrete bridge with the field of experimental evolution. This not only shows that this bridge is possible, but shows the power of Potts models to model it accurately. As the authors focus on how their model can be used to optimize experimental efforts, I see a more deep contribution in evolutionary modeling and its potential to describe or quantify non-trivial evolutionary phenomena. I think the results are substantial, the agreement with orthogonal experiments impressive, and the methodological contributions, like the inclusion of the selection temperature as a fitting parameter, quite innovative. The structure prediction section is also relevant because it provides a convincing hypothesis of two incompatible conclusions in a quite elegant way. Achieving this is not trivial, specially when done in a rigorous way. In conclusion, I think this work is profound, timely and opens up many possibilities for future research. I only have minor comments and questions that might help improve the clarity of this article.

Other than that, I find this work worthwhile sharing with the broad scientific community.

We thank the reviewer for this positive evaluation and the thoughtful comments, which have helped to improve our manuscript.

General comments

Introduction
1) In addition to the work of Fantini et al. and Stiffler et al. that use experimental evolution to provide sequence variability to a global model, there is a similar approach used to understand protein-RNA recognition, as opposed to structure prediction, using a DCA-like approach and high throughput sequencing. If the authors think is relevant I suggest including it as a reference (https://doi.org/10.1038/s41467-018-04729-0).

We thank the reviewer for pointing us to this work, which in fact is very similar in experiment and analysis to the papers by Fantini et al. and Stiffler et al., but using RNA instead of proteins. We have added a paragraph in the Discussion section, pointing out that it might be interesting to explore similar modeling strategies in the case of RNA data.

2) I suggest that the authors use the term Sequence Evolution with Epistatic Contributions (SEEC) to refer to the model introduced by De la Paz et al.

Following the suggestion of the reviewer, we introduced the term at the first reference to De la Paz et al. in the Introduction section, and used SEEC throughout the manuscript when referring to this article.

3) The alignments used in this work are obtained from Pfam which usually only covers a fraction of a given protein as the domain definition across the family is distinct from a specific protein sequence. Could the authors comment on how this fact could affect their model of evolution?

This is an interesting but highly non-trivial question. Based on prior experience with Beta-lactamases (and other proteins, too), it is hard to extend the MSA beyond the Pfam-covered region; e.g. the signal peptide present in the N-terminal region is not well conserved. Extending the MSA therefore typically leads to the inclusion of highly gapped MSA columns, or to the restriction to phylogenetically less diverged protein ensembles. Both tend to limit the accuracy of the resulting mutational landscape, however it may be possible to cover at least some residues outside the Pfam domain.

Beta-lactamases are in the vast majority cases single-domain proteins and therefore the differences between specific proteins are restricted to a limited set of positions in the terminal regions (e.g. TEM-1 and PSE-1). For instance, there are not different domain compositions or important linker regions as it might happen in the case of multi-domain proteins. In addition, the Pfam domain represents well the most

relevant positions shared across the family, which allows us to focus on the most important positions and put under the same framework two divergent proteins such TEM-1 and PSE-1. In this paper, we therefore prefer to directly work with the well-curated Pfam HMM profile. We added a comment on this issue to the Methods section.

Also, for the experimental data from Fantini et al. and Stiffler et al. , did they generate mutational data outside of the domain definition? If so, how did the authors handled these mutated positions outside of the domain definition? Could the authors also comment if they think the model could benefit from a better coverage even if the number of sequences is reduced?

Currently we consider in our analysis only the mutations present within the aligned region and disregard those outside. As proposed by the reviewer and mentioned in our answer to the first part of this question, an extended high-coverage MSA might lead to less sequences, but still to a good description of the local sequence landscape surrounding the experimentally used wildtype sequence, and thus to an improved theoretical modeling of the evolutionary process. However, intuitively, we would expect differences to be minor.

In our work, we did not aim at the most exhaustive and precise model of the evolutionary process, but at a relatively simple but still quantitative scenario. We are convinced that the exploration of more detailed models is important in collaborations we are starting with some experimental colleagues, but that the current purely theoretical paper might lose clarity when exploring these fine details.

4) It is still not clear to me why the in silico MSA does not need reweighting but the experimental does. If the process is modeling the same evolution I would expect that the experimental MSA would also not need reweighting.

The experimental sequences do not have a trivial star phylogeny, so reweighting has a non-trivial effect. As mentioned in the manuscript, our simulated trajectories are independent and all initialized in the wildtype sequence, the simulated phylogeny has therefore a balanced star phylogeny, and reweighting is expected to lead to almost identical sequence weights, i.e. to be without effect on the final result.

We have clarified this point in our manuscript.

Also, line 36 on page 17, mentions an ":auto" parameter for experimental MSAs. Is ":auto" a particular parameter used in GaussDCA? If so, could you give more details? Or if this is just a typo please correct.

The parameter setting ":auto" is an empirical but standard setting in GaussDCA, it calculates the reweighting parameter "theta" as follows: $\min(0.5, 0.1216 / \phi)$ where ϕ is the mean similarity fraction of all pairs of the sequences in the MSA.

We have clarified this point in the manuscript.

5) It would be good if the authors could share their code with the scientific community in a code availability statement.

The manuscript contains a data and code availability statement, which now is updated including the final Github links.

Minor

1. Consider revising this sentence : “The correlated usage of amino acids
in pairs of residue positions can be extracted ..” the term “Correlated usage” is not clear.

We have reformulated the sentence: “The correlations between the amino acids present in pairs of residue positions...”

2. Mixed use of “amino acid” and “amino-acid” , I suggest using “amino acid”

We have used the convention “amino acids” for nouns, and “amino-acid” for adjectives. We have carefully checked our manuscript and removed potential errors.

3. In the last paragraph of page 3, I suggest to change “very efficient” to “efficient” and “very expensive” to “expensive” to keep a more objective tone in the discussion.

We have followed the reviewer’s suggestion.

4. Change “a MSA” to “an MSA”

We have corrected this error.

5. Change “very bad contact prediction” with “inaccurate contact prediction”

We have followed the reviewer’s suggestion.

6. Page 16, methods . Change “amino acid b, which differ” to “”amino acid b, which differs”

We have corrected this error.

Reviewer: 2

In this work, Bisardi et al apply the DCA method to two datasets obtained from experimentally evolved libraries of proteins. The DCA method has become an integral point in protein structure and protein-protein interaction prediction pipelines, relying on sequence covariations to extract information from sequence alignments. In this work, the authors follow up on their previous work of the DCA method

development as well as its application to fitness landscapes. The results are of interest to the community and MBE journal readership. The authors claim the application of the in silico evolution can be used to inform future experiments. I find the article to be of sufficient interest to the community for publication in Molecular Biology and Evolution journal, provided authors can address the points listed below:

We thank the reviewer for the positive evaluation of our manuscript and the helpful suggestions and comments, which we have carefully included into our revision.

1) In the paper, the authors bring up different tools that can be used to infer the J_ij contacts. They mention that the two experimental datasets that they analyzed have been originally analyzed using plmDCA, which is an inferior method in terms of quality of inference to bmDCA and gaussDCA that are used in this work.

In the revised manuscript, we explained better the use of different DCA tools in the manuscript:

- bmDCA is more accurate for the initial landscape inference (from quite abundant and diverged natural homologs), and leads to better results in the evolutionary simulations;
- gaussDCA is less precise but computationally much more efficient. Figures 5 and S7-8 required a huge number (>7000) of contact predictions (each square being an average over 5 independent evolutionary simulations), and the efficiency of gaussDCA was essential for reaching the presented resolution in these figures.

Would the original datasets, reanalyzed with bmDCA (or even adaptive cluster expansion method) perform better and would it be possible to infer contacts from these datasets?

We have reanalyzed the original data from Stiffler et al. (PSE-1) and Fantini at al. (TEM-1) to compare plmDCA and bmDCA in their performance in contact prediction, see the following figures:

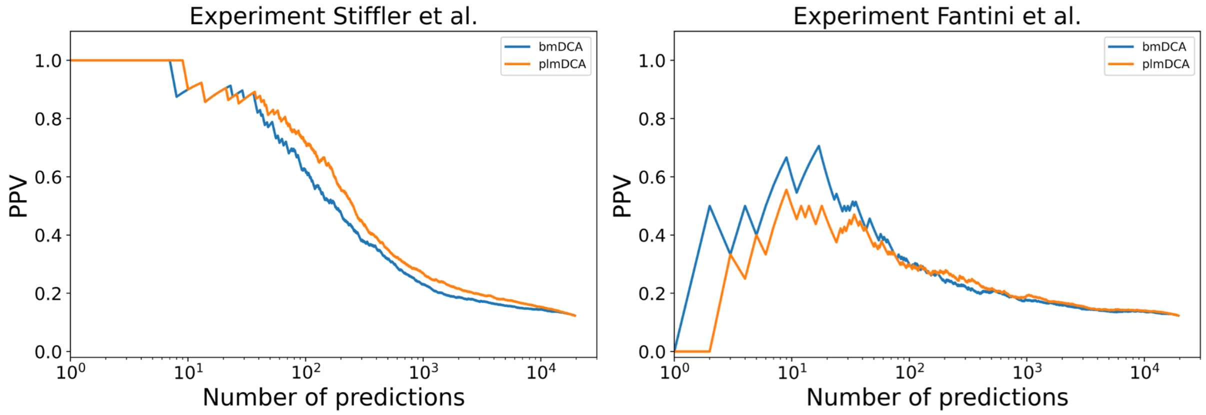

The results are coherent with prior observations in the literature about the relative performance of methods based on pseudo-likelihood maximization or Boltzmann machine learning (including the adaptive cluster expansion, which has a final

refinement using Boltzmann machine learning) – there is only a minor difference between the two. For PSE-1 both give reasonably accurate results, for TEM-1 both detect an inaccurate signal. Since the results are coherent with what is to be expected from the literature, and since the reanalysis of the experimental data is not the objective of our work (as compared to the reproduction and understanding of the observations in terms of evolutionary modeling), we decided to keep our manuscript more focussed and not to include these results into the paper. We hope that the reviewer agrees with this point of view.

2) The authors run a computational experiment where they compare for two different temperatures (corresponding to evolutionary pressure) and number of generated sequences and the divergence of the ensemble in terms of the ability of the models to predict the contacts. They identified a boundary below which there is no sufficient amount of distinct sequences for the models to learn. Is this boundary different for different models (plmDCA, bmDCA and gaussDCA) that are discussed in this work? By how much?

This is a very good, but also complicated question. As mentioned before, the identification of this boundary is computationally very expensive, since a huge number (>7000) of contact predictions have to be performed, partially on very deep MSA. We are therefore constrained to use only GaussDCA in our full analysis.

To provide at least a partial answer, we have analysed the dependence of the accuracy of contact prediction on the sequence number also for plmDCA at two distances from wildtype (i.e., two columns of the full Figure 5A). The results suggest that for small sequence numbers, plmDCA and GaussDCA perform very similarly (low accuracy close to a random prediction), while the improved accuracy of plmDCA over GaussDCA becomes visible only at sufficiently high sequence numbers. At the resolution of our analysis, no shift in the boundary is observable.

Would it be possible to analytically predict this boundary for inference of the Potts models on the sequence ensemble dataset?

This would be great, but unfortunately we have no idea on how to analytically characterize the prediction accuracy for finite MSA. We have added a comment to the conclusions, in the hope that somebody else might have a good idea.

3) The authors judge the quality of the models/datasets by the ability of the trained Potts model to correctly predict pairwise contacts. Is it possible that the Potts model energy landscape is also able to pick up e.g. pairwise “contacts” between residues that might not correspond to actual interaction in the folded protein, but might be either important for its function (e.g. intermediate state during folding, or interaction with its binding partner) and hence even though it might appear as a false positive, it is in fact important for its function?

We agree with the referee, that DCA does not learn only structural contacts, but also some functional or even “phylogenetic” couplings (i.e. spurious couplings induced by phylogeny). However, it has been well established in the literature that the largest

couplings correspond almost exclusively to residue-residue contacts (cf. e.g. Anishchenko et al. 2017, Uguzzoni et al. 2017). In the case of Beta-lactamases, the results on natural data justify the use of contact-prediction accuracy as a quality measure of the models : the MSA of natural homologs leads to 100% or true contacts within the first 100 predictions when using plmDCA (98% for GaussDCA).

A complementary point is that the main objective of our work was to theoretically reproduce the results of the experiments by Stiffler et al. and by Fantini et al., and to understand the differences in their findings. We therefore concentrated on contact predictions, as these two papers did. Indeed it would be interesting, in future work, to have a better look at the biological interpretation of strong couplings that do not correspond to contacts, but this would go beyond the scope of the current work. Another interesting possibility is to investigate the epistasis in functionally linked (although not necessarily close in space) set of residues, e.g. protein sectors and sequence motifs (cf. Reynolds et al. 2016, Tubiana et al 2019, Shimagaki et al. 2019). We have included this point into the Discussion section.

4) Having a nucleotide level mutations (rather than aminoacid level) has been shown to be an important feature of the model in order to be able to fit well the experimental data. Would it further help to also have a parametrized mutation rate (i.e. how many mutations should occur in between selection steps)

We fully agree with the reviewer that our model could have been made more precise, e.g., by introducing a mutation rate and thus multiple simultaneous mutations between the rounds of selection. However, since the experiments performed sequencing only after the selection step, and not after the mutation step, the precise mutation rate cannot be read off explicitly from the data, and would have to be fitted. In our current work, we have therefore chosen the simpler setting, where the mutation rate is effectively parametrized by the number of MCMC steps corresponding to one experimental round, to reach the same Hamming distance in sequence space.

We have clarified this point in the manuscript, when introducing our computational evolutionary model.

5) What I feel a bit lacking in the current version of the manuscript is demonstration of how the proposed model can be used to enrich / guide experimental efforts in the future.
The key steps of relating the modeling to the experiment relies on fitting the model temperature to the slope of DeltaE vs Hamming distance. The model requires some initial set of sequences to be able to fit its temperature, before it can be used to fit to the experimental data and guide the experiment in terms of e.g. the need to turn up or turn down the pressure acting on the sequences.
Could the authors provide an estimate on how many sequences are needed before the model could be fit to infer the corresponding temperature?
Can they run a computational experiment, with the sequences coming from one of the studied datasets, and show if they would be gradually fed sequences obtained

after few round of the experiment, at which point would they be able to fit correctly the temperature and recognize the selection pressure needs to be changed?

These questions are highly interesting and relevant. The reviewer is right that we need experimental data to fit the model temperature corresponding to the applied experimental pressure (e.g., antibiotic concentration). To at least partially answer the reviewer’s question on the basis of the available data, we have done two simple computational experiments based on the PSE-1 data. For each of the rounds 10 and 20, we have subsampled the datasets over a broad range of MSA depths, and fitted the slope for 30 realizations of these subsamples. The following two observations can be made:
(i) the slope can be reliably estimated even for as few as 200-300 sequences, while the accuracy drops when going below 100 sequences (error bars larger than 20% of the slope);
(ii) the slope estimated after 10 rounds is equal to the slope estimated after 20 rounds.
These observations allow for the conclusion that the model temperature can be reliably estimated from small sequence numbers after a few rounds of evolution, so at moderate experimental effort and possibly at different experimental selection pressures. Subsequently, it can be used to simulate much larger experiments. One possible question might be, to find an optimal schedule to diversify sequence as much as possible in a given number of rounds, and at given final fitness.

We have included a more detailed discussion about this point into the Conclusions of our manuscript, and included new supplemental figures for the described computational subsampling experiments. We think, however, that a more detailed computational exploration of evolutionary protocols goes beyond the current slope of the paper, and should be explored in detail in collaboration with experimentalists, as we are starting to do now.
