# Supplementary material for: Modeling Sequence-Space Exploration and Emergence of Epistatic Signals in Protein Evolution
Source: Mol Biol Evol. 2021 Nov 9;39(1):msab321. doi: 10.1093/molbev/msab321 (PMC8789065; doi:10.1093/molbev/msab321)
Supplement: msab321_Supplementary_Data [file msab321_supplementary_data.zip › SI_TEM1evolution.pdf]

Supplementary Information

Modeling sequence-space exploration and emergence of epistatic signals in protein evolution

Matteo Bisardi,[1,2] Juan Rodriguez-Rivas,[2] Francesco Zamponi,[1] and Martin Weigt[2,*]

[1]*Laboratoire de Physique de l'Ecole Normale Supérieure, ENS, Université PSL, CNRS, Sorbonne Université, Université de Paris, F-75005 Paris, France*
[2]*Sorbonne Université, CNRS, Institut de Biologie Paris Seine, Biologie Computationnelle et Quantitative LCQB, F-75005 Paris, France*

[*] correspondence to: martin.weigt@sorbonne-universite.fr

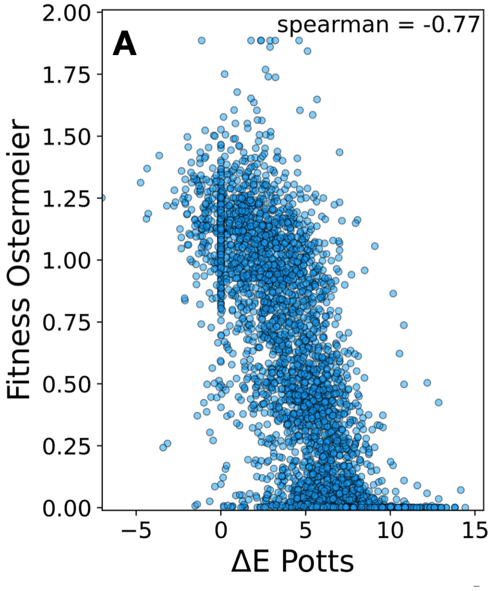

FIG. S1. **Predicted vs. experimentally measured mutational effects for TEM-1:** Scatter plot of the data in Fig. 2 (main text). Panel A shows the experimental results of Ostermeier et al. vs. the DCA predictions using the epistatic Potts model, Panel B vs. the non-epistatic profile model. The Spearman rank correlations between experiments and predictions are displayed in the figures.

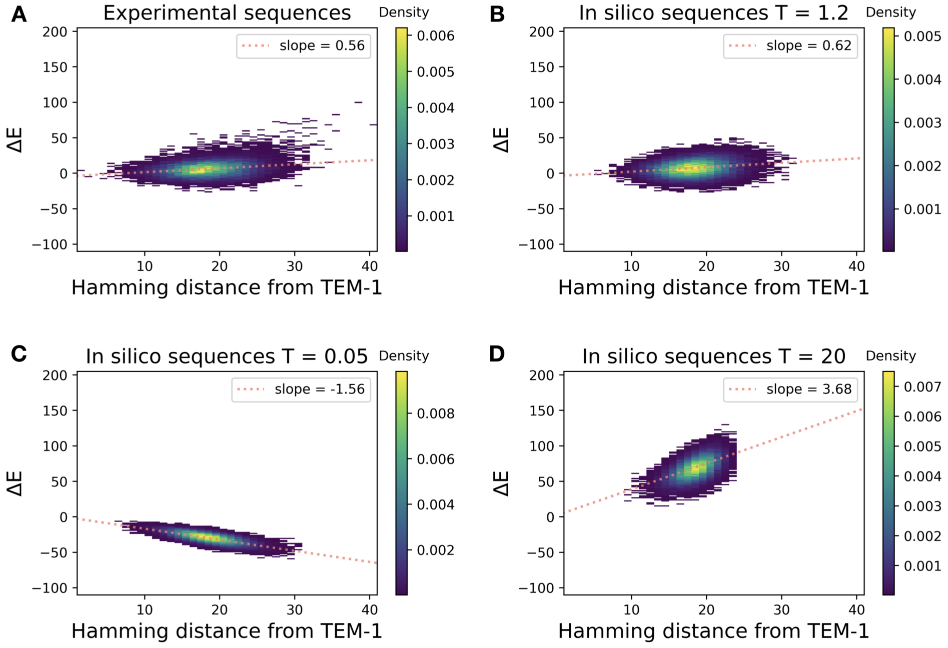

FIG. S2. **Statistical energy in dependence of sequence distance from wildtype TEM-1:** Panel A shows the statistical energies of the sequences from generation 12 in Fantini et al., in dependence of the Hamming distance (number of substituted amino acids) from the wildtype TEM-1. Panel B shows the same quantities for the simulated sequences, where selection strength T and the number of simulated evolutionary steps are adjusted to reproduce the average distance and the slope from Panel A. Panel C shows an example of strong selection ($T \ll 1$) leading to optimized sequences having lower statistical energies / higher fitness. Panel D shows the case of very weak selection ($T \gg 1$) resulting in random, mostly deleterious substitutions strongly increasing statistical energy.

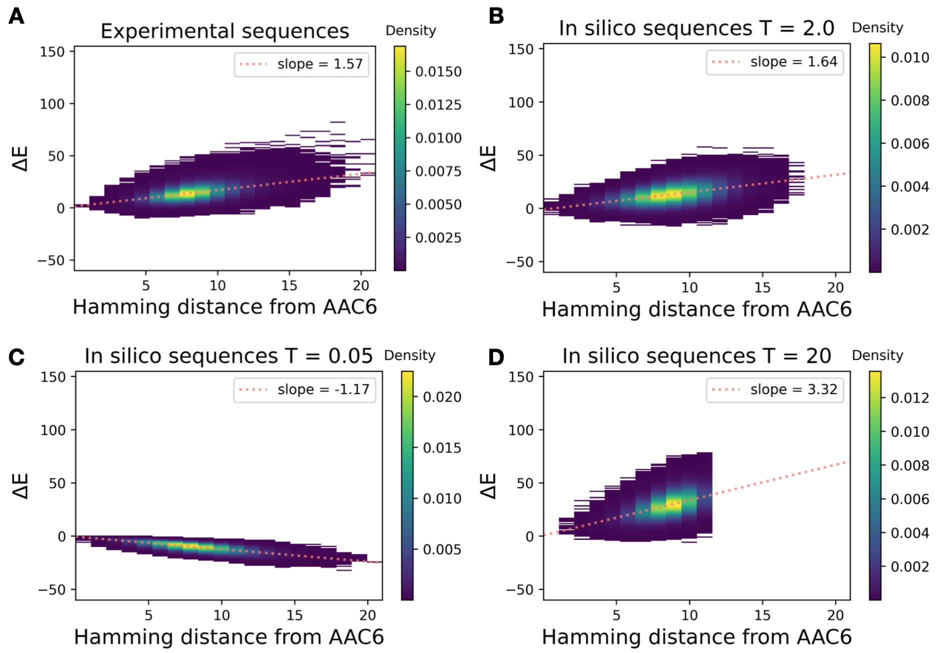

FIG. S3. **Statistical energy in dependence of sequence distance from wildtype AAC6:** Panel A shows the statistical energies of the sequences from round 8 in Stiffler et al., in dependence of the Hamming distance (number of substituted amino acids) from the wildtype AAC6. Panel B shows the same quantities for the simulated sequences, where selection strength T and the number of simulated evolutionary steps are adjusted to reproduce the average distance and the slope from Panel A. Panel C shows an example of strong selection ($T \ll 1$) leading to optimized sequences having lower statistical energies / higher fitness. Panel D shows the case of very weak selection ($T \gg 1$) resulting in random, mostly deleterious substitutions strongly increasing statistical energy.

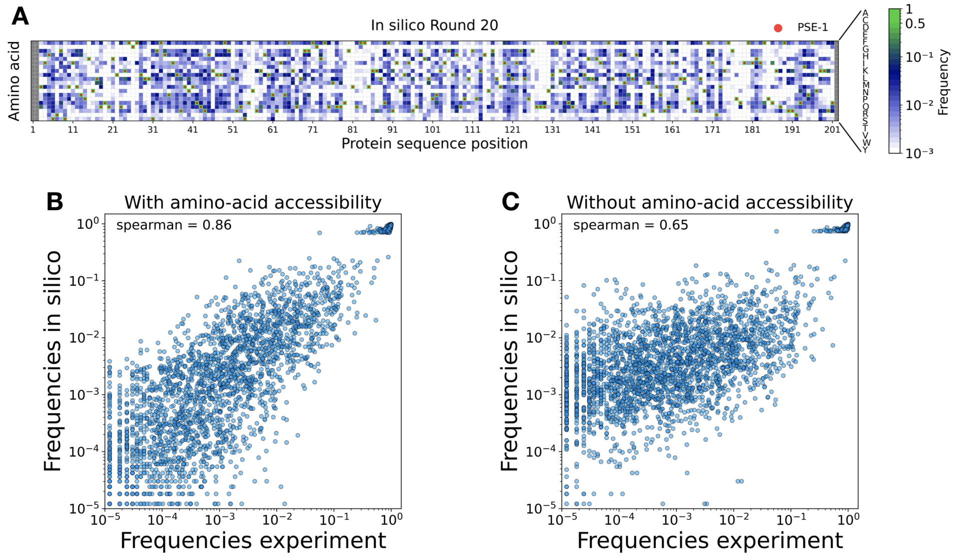

FIG. S4. **Position specific amino-acid frequencies for experimental and simulated sequence libraries for PSE-1:** Panel A shows the frequencies $f_i(a)$ of usage of amino acid a in site i for the simulated sequences without taking into account amino-acid accessibility. Panel B and C show scatter plots of these frequencies for the experimental data vs. simulated data. Panel B takes amino-acid accessibility into account, and shows a higher correlation than Panel C not taking accessibility into account.

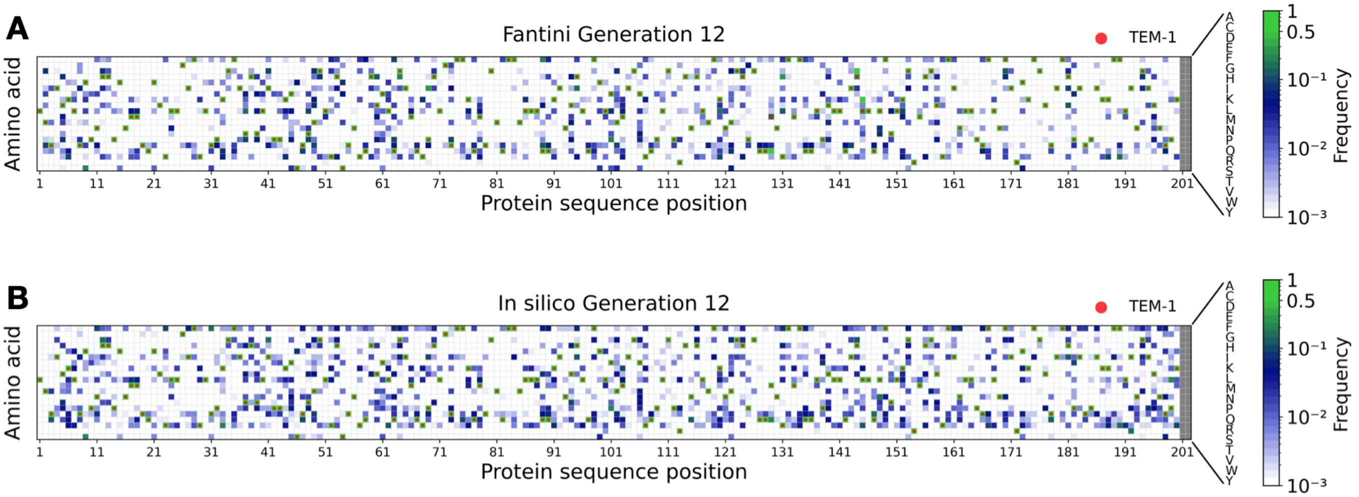

FIG. S5. **Position specific amino-acid frequencies for experimental and simulated sequence libraries:** Panel A shows the frequencies $f_i(a)$ of usage of amino acid a in site i in round 12 of experimental TEM-1 evolution, Panel B shows the same quantity for simulated evolution. Both plots have a Spearman rank correlation of 79%.

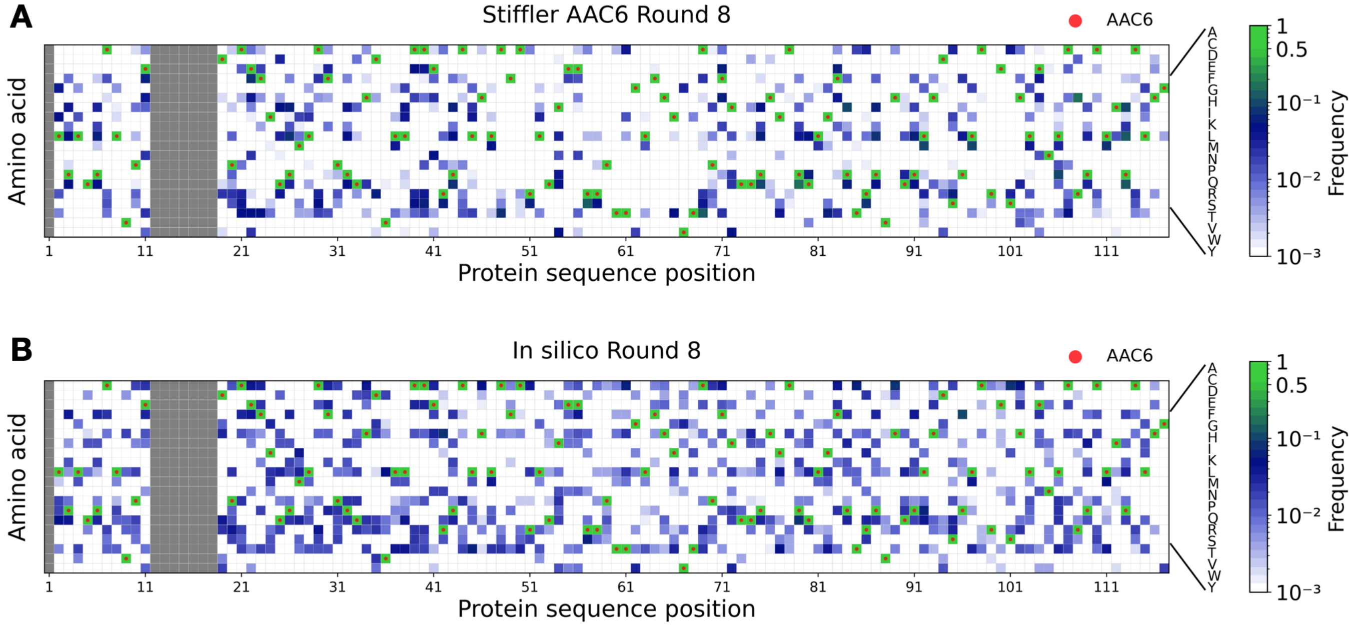

FIG. S6. **Position specific amino-acid frequencies for experimental and simulated sequence libraries:** Panel A shows the frequencies $f_i(a)$ of usage of amino acid a in site i in the experimental AAC6 evolution after round 8, Panel B shows the same quantity for simulated evolution. Both plots have a Spearman rank correlation of 77%.

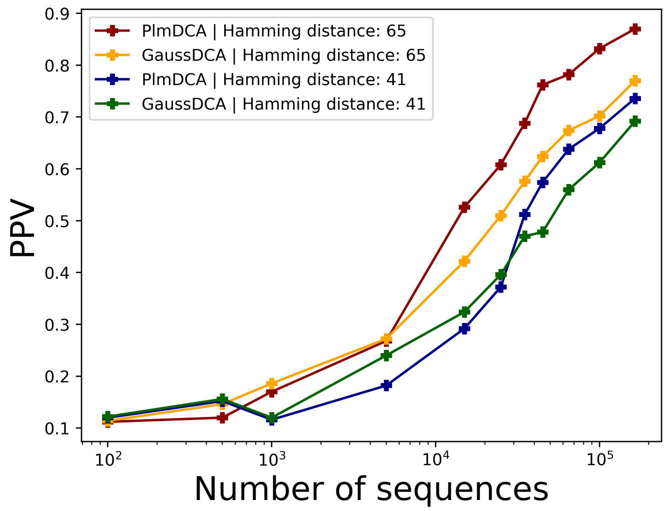

FIG. S7. **Accuracy of contact prediction in dependence of sequence number:** The figure compares the accuracy of contact prediction of plmDCA vs. GaussDCA as a function of the sequence number, for two distances from wildtype PSE-1. The accuracy is measured via the positive predictive value (PPV), *i.e.*, the fraction of true positive contact predictions in the first 100 DCA-predicted contacts, cf. *Methods* for details. The selection strength $T = 1.4$ corresponds to the experimental condition in (Stiffler *et al.* 2020).

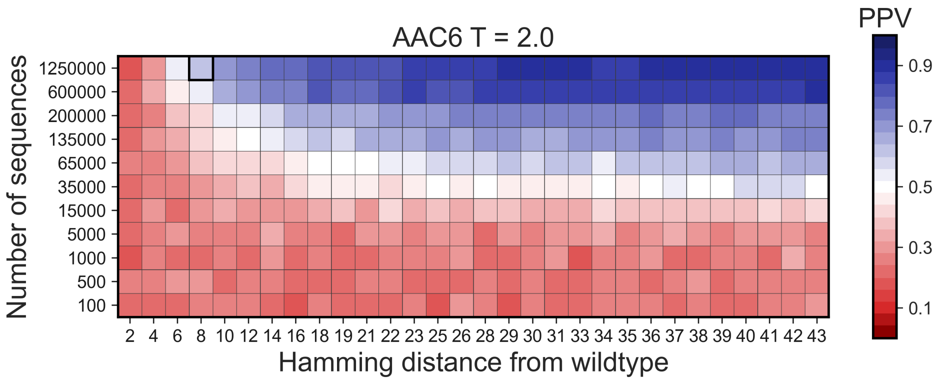

FIG. S8. **Accuracy of contact prediction in dependence of sequence number and sequence divergence:** The figure shows the accuracy of contact prediction as a function of the average sequence divergence from wildtype AAC6 and the depth of the sequenced library, for selection strength $T = 2$. The accuracy is measured via the positive predictive value (PPV), *i.e.*, the fraction of true positive contact predictions in the first 55 DCA-predicted contacts, cf. *Methods* for details. The highlighted square indicates an average Hamming distance of about 8 and a sequence library of 1,250,000, as realized in (Stiffler *et al.* 2020).

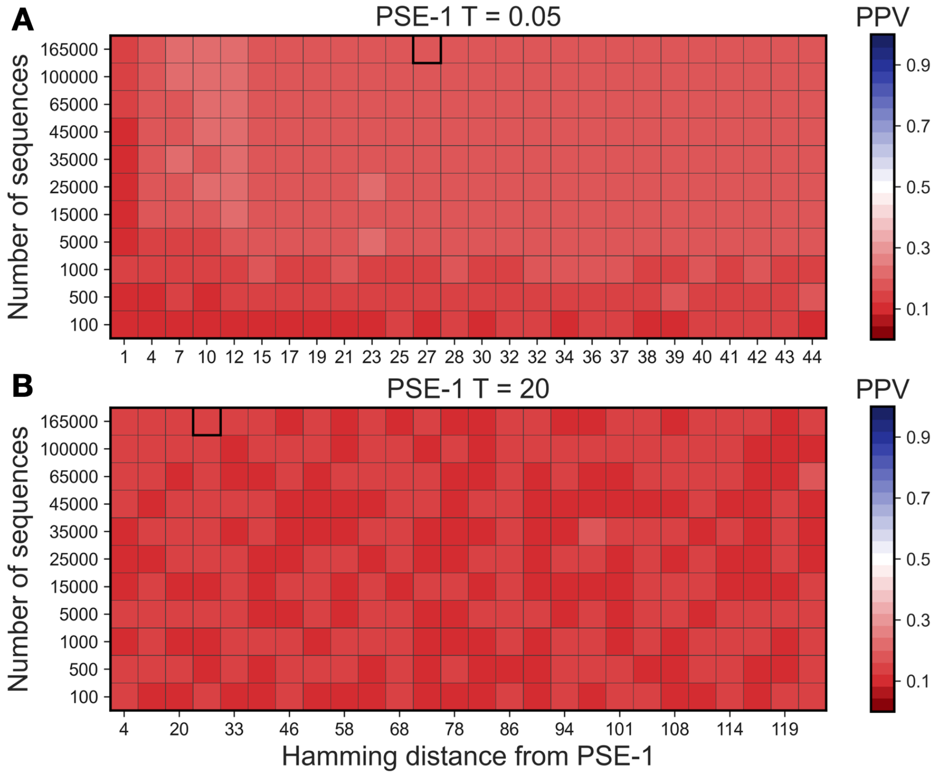

FIG. S9. **Accuracy of contact prediction in dependence of sequence number and sequence divergence:** The panels show, for the case of very strong selection ($T = 0.05$, Panel A) and very weak selection ($T = 20$, Panel B), the accuracy of contact prediction as a function of the average sequence divergence from wildtype PSE-1 and the depth of the sequenced library. The accuracy is measured via the positive predictive value (PPV), *i.e.*, the fraction of true positive contact predictions in the first 100 DCA-predicted contacts, cf. *Methods* for details. The highlighted square indicates an average Hamming distance of about 27 and a sequence library of 165,000, as realized in (Stiffler *et al.* 2020).

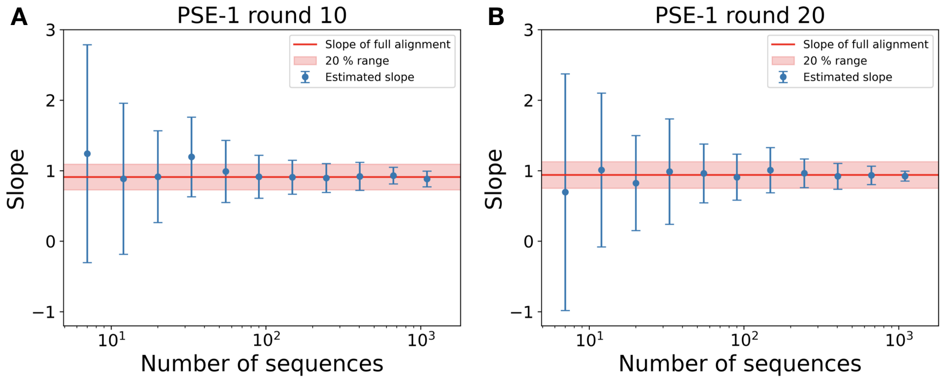

FIG. S10. **Slope of the statistical energy vs. sequence distance from wildtype estimated from subsamples of the PSE-1 sequence libraries:** The panels show the means and standard deviations of the estimated slopes obtained from subsamples of the experimental PSE-1 sequence libraries at round 10 (Panel A) and round 20 (Panel B). The values obtained for the full libraries are evidenced by the red horizontal line, together with a 20%-interval. We observe that estimates fall reliably into this interval when at least 200-300 sequences are used, and that the estimated slopes are almost identical for the libraries obtained after 10 or 20 rounds of experimental evolution in (Stiffler *et al.* 2020).
